# Baseline characteristics for phase II of the Kinshasa Malaria Cohort Study: cohort profile

**DOI:** 10.1136/bmjopen-2024-085360

**Published:** 2024-11-27

**Authors:** Melchior Mwandagalirwa Kashamuka, Kristin Banek, Samuel J White, Joseph Losoma Atibu, Nono M Mvuama, Joseph Alexandre Mavungu Bala, Tommy Mambulu Nseka, Georges Kihuma, Alpha Oumar Diallo, Marthe Nkalani, Georges Emo Mahilu, Faustin Manenga, Goel Varun, Fabien Vulu, Pitshou Nsongo Mampuya, Rhoel Dinglasan, Thierry Bobanga, Michael Emch, Jonathan J Juliano, Jonathan B Parr, Victor Mwapasa, Antoinette Kitoto Tshefu

**Affiliations:** 1 School of Public Heath, University of Kinshasa, Kinshasa, Democratic Republic of Congo; 2 Global and Public Health, Kamuzu University of Health Sciences, Blantyre, Malawi; 3 Institute for Global Health and Infectious Diseases, The University of North Carolina at Chapel Hill, Chapel Hill, North Carolina, USA; 4 Department of Epidemiology, Tulane University Celia Scott Weatherhead School of Public Health and Tropical Medicine, New Orleans, Louisiana, USA; 5 Programme National de Lutte Contre Paludisme, Kinshasa, Democratic Republic of Congo; 6 Department of Epidemiology, The University of North Carolina at Chapel Hill, Chapel Hill, North Carolina, USA; 7 Department of Geography, University of South Carolina, Columbia, South Carolina, USA; 8 Department of Tropical Medicine, University of Kinshasa, Kinshasa, Democratic Republic of Congo; 9 Emerging Pathogens Institute, Department of Infectious Diseases and Immunology, University of Florida, Gainesville, Florida, USA; 10 Department of Geography, The University of North Carolina at Chapel Hill College of Arts and Sciences, Chapel Hill, North Carolina, USA; 11 Division of Infectious Diseases, The University of North Carolina at Chapel Hill School of Medicine, Chapel Hill, North Carolina, USA

**Keywords:** Malaria, EPIDEMIOLOGY, Observational Study

## Abstract

**Abstract:**

**Purpose:**

The Democratic Republic of Congo has one of the highest burdens of malaria in the world, accounting for 12.3% of malaria cases and 11.6% of malaria deaths. The country has been scaling up various malaria control interventions, but how the malaria burden and risk factors have evolved remains unclear. This study aimed to estimate the trend in the burden of malaria in different settings in Kinshasa Province and assess factors contributing to the infection.

**Participants:**

From 2018 to 2022, a cohort study was conducted in three health areas in Kinshasa Province known to have varying malaria endemicities: Voix du Peuple (urban), Kimpoko (peri-urban), and Bu (rural) health areas in Kinshasa Province. Participants 6 months of age and older were recruited and followed biannually through household visits and encouraged to visit study health centres whenever they felt ill. The participants’ sociodemographic data, illness history and access to malaria interventions were collected along with blood specimens. During the same study period, mosquitoes and larvae were collected to study entomological characteristics that may contribute to malaria transmission.

**Findings:**

A total of 1635 participants were recruited from 239 households. The median number of participants per household was 6 (IQR, 5–9). Over half (54.7%) of the participants were female, 14.6% were under-5 children, and 33.3% were 5–14 years old. Household net ownership and reported use the previous night were 77.8% and 79.1%, respectively. Overall, malaria prevalence by rapid diagnostic test at baseline was 33.4%. Of the recruited participants, 1390 (85%) completed the 4 years of follow-up.

**Future plans:**

Planned analyses include calculating malaria prevalence and incidence trends at follow-up as well as risk factors for infection. Continued analyses will link epidemiological and entomological data to understand malaria transmission evolution and the sustained high prevalence of malaria over time in various settings.

**STRENGTHS AND LIMITATIONS OF THIS STUDY**

- This cohort study compares malaria transmission across seasons and multiple epidemiological settings in the Democratic Republic of Congo (DRC) in the context of increasing coverage of malaria control interventions.
- The prospective design-linking malaria epidemiological and entomological data are a major strength of this study.
- The study is subject to selection and information bias due to its observational nature.
- The passive surveillance system (health facility visits) may not have comprehensively captured acute illnesses, as some participants likely sought care for fever elsewhere.

## Introduction

 The Democratic Republic of Congo (DRC) accounts for 12.3% of the world’s annual malaria cases and 11.6% of malaria deaths.^1^ To reduce the malaria burden in the DRC, the National Malaria Control Program (NMCP) has scaled up interventions and management measures by increasing long-lasting insecticidal nets coverage, as well as the availability and use of rapid diagnostic tests (RDTs) and artemisinin-based combination therapies (ACT).^2 3^ Yet, despite increased coverage of these interventions, prevalence remains high, with limited information about local behavioural and environmental determinants that drive the sustained high disease burden in Kinshasa, DRC.^4 5^

Malaria prevalence and incidence estimates in the DRC come from surveys (eg, Demographic and Health Surveys (DHS) and the Multiple Indicator Cluster Surveys)^6^,^8^ or aggregated routine health facility data through the District Health Information Software (DHIS2). Although the national surveys provide nationally representative data, they are conducted infrequently (4–5 years), providing only snapshots of malaria prevalence and risk factors at one point in time. The health facility data are also limited because they capture counts of malaria episodes among those seeking treatment at health facilities, which represents only 38% of malaria cases.^9^ Additionally, national surveys and routine data do not capture age-specific data on children aged 5–14 years, despite documentation that this age group has the highest disease burden and may contribute significantly to malaria transmission.^10 11^ Indeed, previous DHS surveys (2007 and 2013–14) and the ongoing DHS 2023–2024 have excluded the age group 5–14 years. As malaria data from the DHIS2 are aggregated into only two groups, 0–5 years and over 5 years, they also do not provide the age group data needed for targeted malaria control programming. By 2023, only one cross-sectional study in Kinshasa had explored malaria transmission dynamics in school-age children that showed low allelic diversity and multiplicity of infection of *P. falciparum*, which reflects a moderate intensity of malaria transmission in those study areas. ^12^

An understanding of local malaria burden and distribution and transmission dynamics is needed to improve malaria control programme planning. Likewise, a better understanding of host, vector and parasite interactions in different environments that may exacerbate malaria infection is needed. Longitudinal studies offer an opportunity to assess malaria transmission dynamics over time by combining data on malaria infection and disease rates (prevalence and incidence rates) across different age groups with entomological indicators and parasitological parameters.^13^ While there have been many longitudinal epidemiological and entomological studies in sub-Saharan Africa, few combine entomology and malaria epidemiology, especially in settings of varying transmission intensity.^14 15^

To better characterise the malaria trends and transmission dynamics in DRC and to generate data for action in order to meet the Sustainable Development Goal target 3.3 to reduce malaria case incidence and malaria mortality by at least 90%,^16^ we conducted a 4-year longitudinal cohort study combining malaria epidemiological and entomological data across different malaria endemicities and age groups in Kinshasa Province, DRC. Additionally, we incorporated molecular methods to identify insecticide resistance in the mosquitoes,^17^ drug resistance in the parasites,^18^ and the presence or absence of *P. falciparum* histidine-rich protein 2 (*pfhrp2*) gene deletions, all of which challenge malaria control efforts.^19 20^ This paper describes the baseline characteristics of the cohort and study sites, which provide a benchmark for understanding the evolution of malaria infection, transmission and risk factors of infection that can be targeted by interventions to improve control and, ultimately, eliminate malaria in the DRC.

## Cohort study

This was a prospective observational cohort study comprising 1635 individuals from 7 sites in Kinshasa.

### Study sites

**Figure 1:**
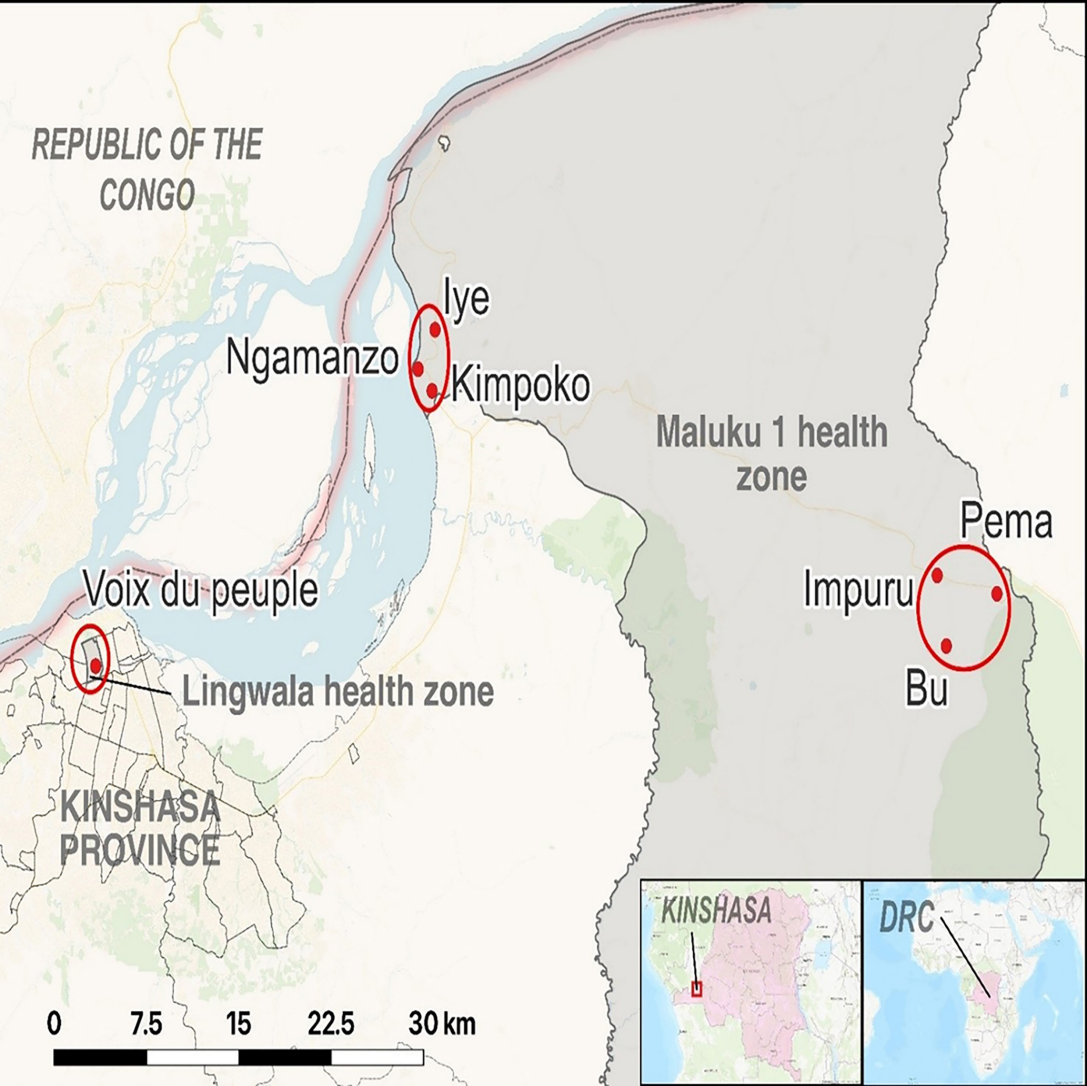
Location of the seven study sites within the two health zones.

We conducted the study in seven study sites located in three health areas in Kinshasa Province. Two health areas were in the Maluku 1 health zone (Bu and Kimpoko), and one was in the Lingwala health zone (Voix du Peuple), selected in phase 1. The two health zones included in this study had differing ecological characteristics. These health areas represent different settings and malaria transmission intensities: Bu (rural/high), Kimpoko (peri-urban/moderate) and Voix du people (urban/low). Maluku 1 has marsh/swamp areas near the Congo River, where the Kimpoko health area is located, and the Bateke plateau, where the health area Bu is located, with a giant ancient sand dune system covered by large grass and wooded savanna patches separated by fine lines of dense gallery forest, and two river valleys. In Maluku 1, the population size on average was 10 654 people per health area, distributed across 26 villages ~1522 households. In contrast, the Lingwala health area is urban, with an average population size of 16 336 people and 2047 households per health area (figure 1).

### Cohort description

This prospective observational cohort study (phase II) was a continuation of a malaria cohort study established in 2015 (phase I) in the same study sites. Participant characteristics were compared between the two phases and are described in online supplemental file 1. The primary objective of the phase I study was to understand the relative contribution of individuals with subpatent malaria parasitemia to the overall transmission reservoir as measured by gametocytaemia over time at varying levels of endemicity across all ages.^21 22^ Phase II enabled the continued collection of malaria epidemiological data alongside entomology data to better understand how human and mosquito factors affect malaria transmission to describe malaria incidence according to seasonality and varying sites in these same health areas.

### Study population

All households that participated in phase I (2015–2017) were eligible to participate in phase II. We enrolled permanent household residents aged ≥6 months who provided written informed consent to participate in the 4-year period (2018–2022). We excluded household members who were unable to provide informed consent, did not understand languages used by study team members or were seriously ill. Using an estimated malaria prevalence of 20%, participant loss-to-follow-up of 15%, and 90% power to detect an increase in prevalence to 25% (alpha=0.05) when comparing outcomes between time points, the target sample size was estimated to be 1600.

### Study overview

**Figure 2:**
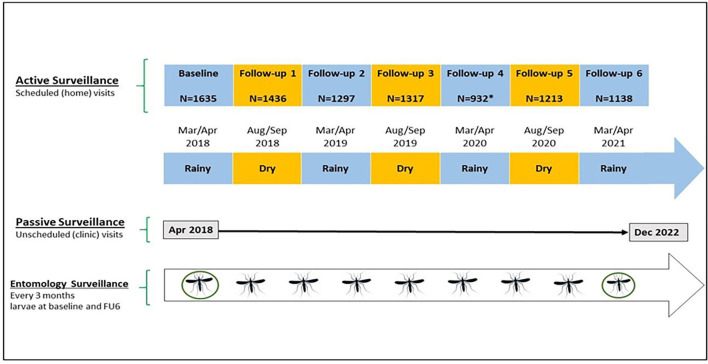
Study overview. The figure depicts the three types of data collection over the study period 2018–2022: active, passive and entomological surveillance. Blue-shaded boxes represent the rainy season. Yellow-shaded boxes represent the dry season. Mosquito icons represent mosquito catches. Circled mosquitoes represent larvae collection. FU6, follow-up 6.

Participant enrolment occurred between March and October 2018. Table 1 and figure 2 summarise the timing and type of data collected. Malaria epidemiological data were collected actively (at the household level) and passively (at the clinic level). Active surveillance data were collected twice annually (wet and dry season) during scheduled household visits. Passive surveillance occurred at local study clinics and included all study-enrolled participants seeking treatment for fever. Entomological surveillance occurred nine times throughout the study and involved interior and exterior mosquito catches.

**Table 1:** Collection of epidemiological, laboratory and entomological data for the cohort longitudinal study

	Baseline	Follow-up1	Follow-up2	Follow-up3	Follow-up4	Follow-up5	Follow-up6
Variables							
Active surveillance*							
Demographic	X	X					
Socioeconomic	X	X					
Occupation	X	X					
Clinical	X	X	X	X	X	X	X
Travel history	X	X	X	X	X	X	X
Malaria RDT	X	X	X	X	X	X	X
Malaria microscopy	X	X	X	X	X	X	X
PCR *Plasmodium falciparum* infection	X	X	X	X	X	X	X
Passive surveillance†							
Clinical	X	X	X	X	X	X	X
Malaria RDT	X	X	X	X	X	X	x
Malaria microscopy	X	X	X	X	X	X	X
PCR *P. falciparum* infection	X	X	X	X	X	X	X
Haemoglobin	X	X	X	X	X	X	X
Entomology surveys‡							
Species identification	X	X	X	X	X	X	X
*P. falciparum* infection Elisa	X	X	X	X	X	X	X
PCR *Anopheles gambiae* species	X	X	X	X	X	X	X
Phenotypic insecticide resistance	X						X

* Variables collected during the seven household visits in enrolled households.

† Variables related to the passive surveillance collected at the study health area.

‡ Variables regarding the entomology in designated five households in each study.

RDTrapid diagnostic test

### Data collection process

#### Site selection and household sampling

Criteria for the site selection, household sampling and subjects have been reported previously.^21^,^23^ In summary, for each of the seven surveillance locations, two geographical coordinates within the site, a beginning and end point, were selected. Households were then randomly recruited, starting from the household nearest the start point coordinate and moving towards the household nearest the end point coordinate. Geographical coordinates of households that consented to participate in the study were recorded using GPS devices (Garmin Dakota 20 GPS). Household enrolment at the start of the phase II study is described below. In the DRC, a household is defined as a person or group of persons who live together in the same house or compound, share the same housekeeping arrangements and are catered for as one unit, meaning they commonly provide and share food.

Household sampling for entomological surveillance was conducted to ensure a spatially representative coverage of the households enrolled in each site. Specifically, using the GPS coordinates and household IDs of enrolled households, each site was divided into five groups with an equal number of households in each group, and then one household from each group was randomly selected to participate in entomological surveillance. If the household declined to participate in entomological surveillance, it was replaced by the next closest household in that group. In total, 35 sentinel households (five by study site) participated in the entomological surveillance arm of the study. Onlinesupplemental files 2  3 provide a detailed description of the household and entomological sampling design.

#### Fieldworker and community health worker training

To ensure that data collection would move smoothly, all personnel involved in the study, including the core team, interviewers, laboratory technicians and study health centre personnel, were trained in the study design, research ethics, interviewing techniques and specimen collection. A pilot test was performed before actual data and specimen collection. Community health workers (CHWs) were also trained to ensure that they had an idea about the purpose of the study, basic notions of research ethics and malaria symptoms to have arguments when encouraging participants to visit study health centres anytime they felt sick.

#### Enrolment

Field teams comprised two persons: one interviewer and one laboratory technician. During the first household visit (Baseline), the interviewer initiated the recruitment and enrolment process with the head of the household, either the father, the mother, or the legal designate, who confirmed the eligibility criteria for each participant and obtained consent or assent before administering the household questionnaire, that captured sociodemographic characteristics of all household members, including name, age and sex, education, occupation, religion and their relationship with the head of the household. Each enrolled household member received a study card, which study team members used to confirm the household member’s enrolment status during future household active surveillance or study clinic visits (passive surveillance).

#### Active surveillance data collection

Field teams visited households twice yearly, once in the rainy and once in the dry seasons. For each visit, the team administered a comprehensive set of questions to each participant to ascertain previous malaria symptoms, antimalarial use, bednet ownership and utilisation and travel history included a comprehensive set of questions related to previous malaria symptoms, antimalarial use, bednet ownership and utilisation and travel history. Teams also collected blood samples from finger-prick or heel-prick (young infants), which were tested for malaria using an RDT according to manufacturer instructions (baseline: SD Bioline Ag *P.f*./Pan RDT (05FK60), Alere, Gyeonggi-do, Republic of Korea, or follow-up visits 1–6: CareStart Malaria Pf HRP2 Ag (02571), Access Bio, Somerset, New Jersey) and read by the well-trained field team members. RDT-positive persons were referred for treatment at the local study clinic and followed to assess whether treatment was received. Treatment was provided according to the national malaria treatment guidelines.^24^ Additionally, the collected blood samples were tested by microscopy later in the Kinshasa School of Public Health laboratory in Kinshasa and conserved using dried blood spots (DBS) for future molecular testing.

#### Passive surveillance data collection

A nurse and laboratory technician were employed in each study clinic in the three study health areas. If study participants visited the clinic with febrile illness (>37.5°C), the study nurse captured signs and symptoms, diagnostic (RDT, microscopy and haemoglobin.) relevant data (temperature, RDT, microscopy and haemoglobin), and prescribed treatment on paper study forms and obtained a DBS for future molecular testing. Participants with positive RDT results were treated at the study clinic according to the national malaria treatment guidelines. When participants experienced signs of severe malaria, they were referred to the study hospital. An expert microscopist prepared and read all microscopy slides for asexual parasite density, gametocyte densities and *Plasmodium* species. Haemoglobin was measured using the HemoCue Hb 201+System (HemoCue AB, Ängelholm, Sweden). Study supervisors collected forms and biological samples biweekly and brought them to the study office in Kinshasa for processing, quality control, data entry and storage.

#### Entomological data collection

At each entomological study household visit, we collected for 3 consecutive days at five households in each of the seven study sites using the United States Center for Disease Control light traps and pyrethrum spray catch.^25^ Adult female anopheline mosquitoes caught at the field sites underwent a panel of tests, microscopic species identification and ELISA for *Pf* infection. Mosquitoes identified morphologically as *Anopheles gambiae sensu lato* were analysed by PCR restriction fragment length polymorphism to determine subspecies. Larvae were collected from circumstantial breeding sites across all seven locations during the rainy seasons at baseline and again at the final follow-up visit (follow-up 6). Larvae were reared to adult mosquitoes and tested for insecticide resistance.

#### Adverse event data collection

We instructed participants to report to the local health centre (study clinic) if they experienced any adverse event related to the study procedures or developed signs of severe disease. Adverse events were recorded and reported to the study team at monthly supervision visits. All serious adverse events, including severe disease, referrals and deaths, were reported immediately to the study supervisory team and the different Institutional Review Boards (see ethics section). The study team worked with the NMCP through SANRU (Santé Rurale) Asbl, a non-governmental organisation and major NMCP implementing partner, and the health zones where our sites were located to ensure that all study clinics were adequately stocked with RDTs, microscopy supplies and ACTs (artemether-lumefantrine and/or artesunate-amodiaquine).

### Data management and statistical analyses

Data for baseline, active surveillance follow-up visits 1–3, and all passive surveillance visits were collected on paper forms and double-entered into an online Research Electronic Data Capture (REDCap) database hosted by the University of North Carolina at Chapel Hill.^26 27^ We used REDCap Mobile via android tablets to collect data for the active surveillance follow-up visits 4–6, and data were validated for accuracy before uploading to the REDCap server.

Descriptive analyses of the baseline sociodemographic and clinical variables were conducted in SAS software V.9.4 (SAS Institute). Frequencies and proportions were calculated for categorical variables, and mean, median, SD and IQR were calculated for continuous variables. χ^2^ and t-tests will be used to investigate associations between endemicity, gender, age categories and malaria infection.

### Patient and public involvement

Study participants and the public were not involved in the study design, setting of research questions, or recruitment of participants, but their continued engagement and participation have been instrumental in implementing this study. Several community engagement activities were conducted to gain insight into the participant experience and encourage continued participation Engagement activities were primarily conducted through door-to-door household visits by CHWs, some of which were also study participants. Additionally, informational and dissemination meetings with community leaders and heads of households took place before and during implementation. Final findings will be disseminated across study communities through community meetings, CHWs, heads of households and opinion leaders. Furthermore, preliminary results were presented and discussed with scientists and stakeholders at national malaria meetings, and the final results will be presented to the NMCP for decision-making purposes.

### Baseline findings

#### Participant recruitment and characteristics

**Figure 3:**
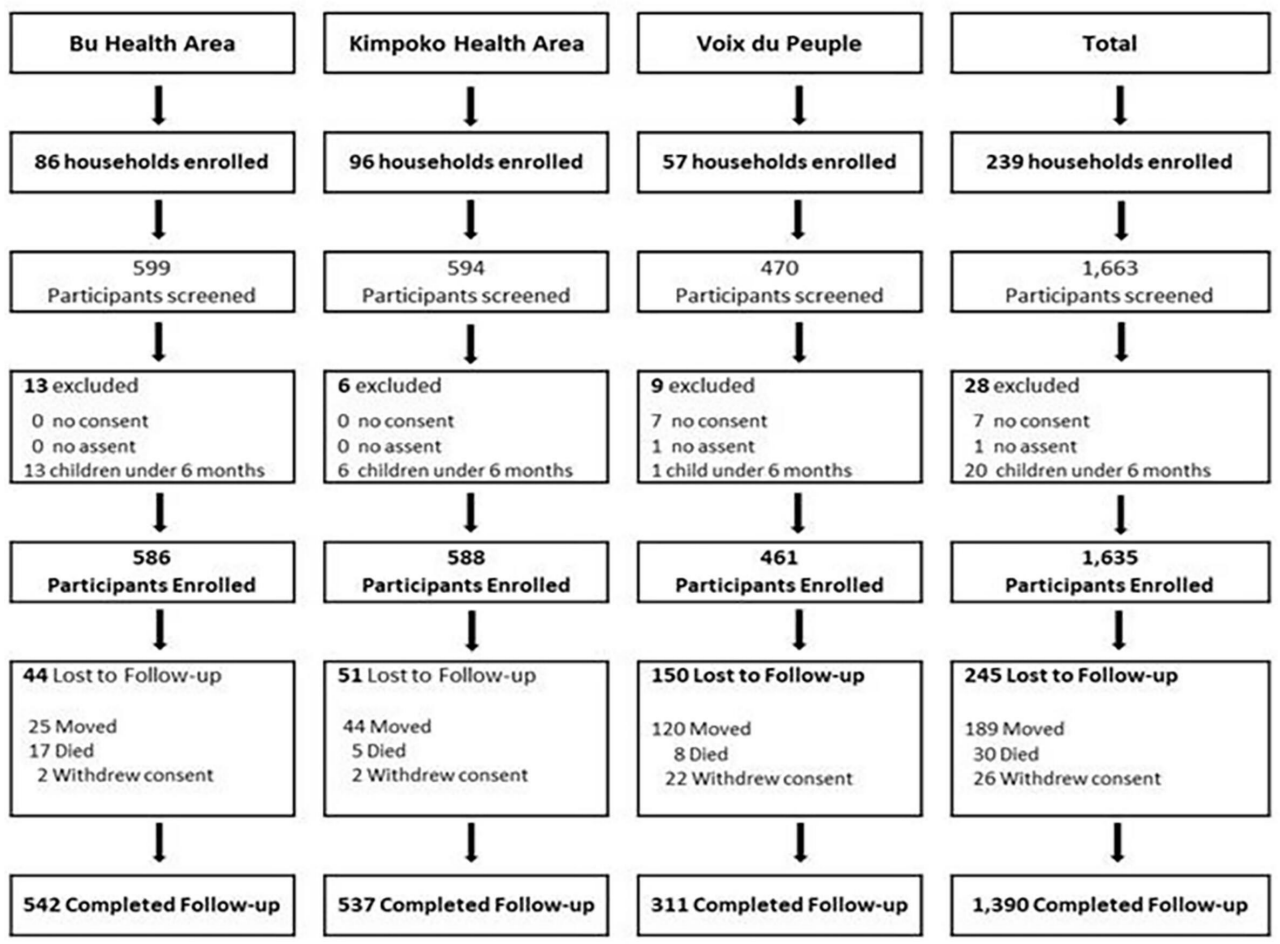
This figure represents the participant flow from the enrolment at baseline to the study’s last completed household visit. Participant retention was 85%.

A total of 239 households were enrolled between March and October 2018: 86 in Bu, 96 in Kimpoko, and 57 in Voix du Peuple health areas, comprising 1635 enrolled participants (586 in Bu, 588 in Kimpoko, and 461 in Voix du Peuple). The enrolled participants included 1014 (64%) participants from Phase I of the project and 621 newly recruited participants to meet the minimum target sample size (n=1600). After 4 years of follow-up, 245 (15.0%) participants were lost (figure 3). This loss of follow-up was caused essentially by households and participants’ movements, participant withdrawal of consent, or death. As expected, the urban site (Lingwala) had the highest proportion of participant loss of follow-up, 150 out of 245 (61%), mainly in terms of movements, probably because of high unemployment, as shown in table 2. Similarly, we had a greater loss of follow-up during the COVID-19 pandemic. It should be noted that movement restrictions due to the COVID-19 pandemic impacted data collection; thus, the active household surveillance visit was not conducted in Lingwala in March–April 2020, as shown in the study overview (figure 2).

**Table 2:** Cohort phase II baseline participant characteristics

	Bu	Kimpoko	Voix de peuple	Total
Number of participants enrolled	586	588	461	1635
Female	326 (55.6%)	303 (51.5%)	265 (57.5%)	894 (54.7%)
Pregnant women*	8/154 (5.2%)	7/149 (4.7%)	4/160 (2.5%)	19/463 (4.1%)
Age group				
<5	121 (20.7%)	80 (13.6%)	38 (8.2%)	239 (14.6%)
5–14	225 (38.4%)	210 (35.7%)	110 (23.9%)	545 (33.3%)
15+	240 (41.0%)	298 (50.7%)	313 (67.9%)	851 (52.1%)
Marital status†				
Never married	42 (17.5%)	102 (34.2%)	172 (55.0%)	316 (37.1%)
Married or living together	159 (66.3%)	150 (50.3%)	78 (24.9%)	387 (45.5%)
Divorced/separated	26 (10.8%)	14 (4.7%)	16 (5.1%)	56 (6.6%)
Widow/widower	11 (4.6%)	14 (4.7%)	31 (9.9%)	56 (6.6%)
Missing	2 (0.8%)	18 (6.0%)	16 (5.1%)	36 (4.2%)
Education‡				
None	55 (12.0%)	37 (7.3%)	10 (2.4%)	102 (7.3%)
Any primary school	237 (51.0%)	182 (35.8%)	79 (18.7%)	498 (35.7%)
Any secondary school	147 (31.6%)	235 (46.3%)	215 (50.8%)	597 (42.8%)
University or higher	3 (0.7%)	9 (1.8%)	86 (20.3%)	98 (7.0%)
Other	2 (0.4%)	0 (0.0%)	1 (0.2%)	3 (0.2%)
Missing	21 (4.5%)	45 (8.9%)	32 (7.6%)	98 (7.0%)
Income-generating activities†				
No occupation	62 (25.8%)	64 (21.5%)	107 (34.2%)	233 (27.4%)
Government official	10 (4.2%)	27 (9.1%)	17 (5.4%)	54 (6.4%)
Soldier or policeman	3 (1.3%)	0 (0%)	0 (0%)	3 (0.4%)
Private company employee	1 (0.4%)	4 (1.3%)	16 (5.1%)	21 (2.5%)
Farmer or fisherman	35 (14.6%)	34 (11.4%)	0 (0%)	69 (8.1%)
Driver	3 (1.3%)	3 (1.0%)	1 (0.3%)	7 (0.8%)
Vendor	15 (6.3%)	42 (14.1%)	43 (13.7%)	100 (11.8%)
Small personal business	4 (1.7%)	5 (1.7%)	6 (1.9%)	15 (1.8%)
Student	9 (3.8%)	17 (5.7%)	67 (21.4%)	93 (10.9%)
Other	85 (35.4%)	59 (19.8%)	35 (11.2%)	179 (21.0%)
Missing	13 (5.4%)	43 (14.4%)	21 (6.7%)	77 (9.1%)
Religion				
Evangelical	206 (35.2%)	217 (36.9%)	256 (55.5%)	679 (41.5%)
Protestant	116 (19.8%)	124 (21.1%)	21 (4.6%)	261 (16.0%)
Catholic	108 (18.4%)	50 (8.5%)	94 (20.4%)	252 (15.4%)
Kimbaguist	11 (1.9%)	35 (6.0%)	21 (4.6%)	67 (4.1%)
Muslim	8 (1.4%)	10 (1.7%)	0 (0.0%)	18 (1.1%)
Traditional religion	1 (0.2%)	4 (0.7%)	1 (0.2%)	6 (0.4%)
Other	122 (20.8%)	111 (18.9%)	40 (8.7%)	273 (16.7%)
Not religious	5 (0.9%)	3 (0.5%)	4 (0.9%)	12 (0.7%)
Missing	9 (1.5%)	34 (5.8%)	24 (5.2%)	67 (4.1%)

* n=463 women of childbearing age 15+.

† Only collected for participants 15 years and older (n=851).

‡ Only collected for participants 5 years and older (n=1396).

**Table 3:** Cohort phase II household characteristics (n=239)

	Bun (%)	Kimpokon (%)	Voix du PeupleN (%)	Totaln (%)
Number (%) of HHs	86 (36.0%)	96 (40.2%)	57 (23.9%)	239
Median number participants per household (IQR)	7 (4–9)	6 (4.5–8)	8 (6–11)	6 (5–9)
Wealth quintile				
1 (Poorest)	30 (34.9%)	18 (18.8%)	0 (0%)	48 (20.1%)
2	30 (34.9%)	17 (17.7%)	0 (0%)	47 (19.7%)
3	22 (25.6%)	26 (27.1%)	0 (0%)	48 (20.1%)
4	4 (4.7%)	33 (34.4%)	11 (19.3%)	48 (20.1%)
5 (Least poor)	0 (0%)	2 (2.1%)	46 (80.7%)	48 (20.1%)
Household net ownership	66/86 (76.7%)	78/96 (81.3%)	42/57 (73.7%)	186/239 (77.8%)
Median number of nets (IQR)	2 (1–3)	2 (2–3)	3 (2–3)	2 (1–3)
Number of nets per household	(1, 6)	(1, 4)	(1, 6)	(1, 6)
0	20 (23.3%)	18 (18.8%)	15 (26.3%)	53 (22.2%)
1	26 (30.2%)	15 (19.2%)	7 (16.7%)	48 (25.8%)
2	23 (26.7%)	22 (28.2%)	13 (31.0%)	58 (31.2%)
3	14 (16.3%)	24 (30.8%)	13 (31.0%)	51 (27.4%)
4	1 (1.5%)	9 (9.4%)	4 (9.5%)	14 (7.5%)
5	1 (1.5%)	0 (0%)	3 (7.1%)	4 (2.2%)
6	1 (1.5%)	1 (1.3%)	2 (4.8%)	4 (2.2%)
Unspecified number	0 (0%)	7 (9.0%)	0 (0%)	7 (3.8%)
Net source* (n=186)				
Campaign	49 (74.2%)	51 (65.4%)	34 (81.0%)	134 (72.0%)
Health facility or prenatal consult	15 (17.4%)	8 (8.3%)	5 (8.8%)	28 (15.8%)
Bought	1 (1.5%)	10 (12.8%)	2 (4.8%)	13 (7.0%)
Other	1 (1.5%)	2 (2.6%)	0 (0%)	3 (1.6%)
Unknown	0 (0%)	7 (9.0%)	1 (2.4)	8 (4.3%)
Mean % of Nets used in the household the previous night	73.9%	88.7%	71.5%	79.1%

* Percentages calculated based on the total number of households with any net (n=182).

Tables2  3 show the baseline characteristics of the households and study participants, respectively. The median number of enrolled participants per household was 6.0 (IQR=5–9). Over half of the participants (54.7%) were female (table 3). Children aged <5 years represented 14.6% of the study population, and 33.3% were school-aged children 5–14 years of age. As expected, the urban site had a low percentage of this age category, which may be due to the fact that family planning tends to be three times higher among urban than rural women. It has also been demonstrated that educated and wealthy people tend to control birth. Indeed, as expected, the urban site participants were more educated and less poor compared with the peri-urban and rural populations (tables2  3).

Most study households (77.8%) owned at least one mosquito net. The median number of long-lasting insecticidal nets per household was 2 (IQR: 1–3). Net use was high, with 79.1% of nets reported being used the night before the interview. Kimpoko had the highest rate compared with Bu and Voix du Peuple. This may be due to the high number of mosquitoes in Kimpoko. Indeed, the Human Biting Rate and the Entomological Inoculation rate were higher in Kimpoko compared with Bu and Voix du Peuple (data not yet published). The primary net source was mass distribution campaigns (72.0%). The overall malaria prevalence by RDT at baseline was 33.4% (Bu: 60.5%, Kimpoko: 37.1% and Voix du Peuple: 2.4%). Baseline malaria prevalence was highest for participants aged 5–14 years old (52.6%), followed by participants aged ≥15 years (31.2%) and children aged <5 years (16.2%).

### Ongoing activities and future plans

All active surveillance household visits (baseline and six follow-up visits) were completed in May 2021, and database validation was completed by November 2022. Passive surveillance visit data collection was completed in December 2022, and data entry was completed in September 2023. The study team will analyse active and passive surveillance results to determine malaria prevalence and incidence trends by age in varying endemicity settings as well as across seasons (dry vs rainy). Entomology data on *Anopheles* mosquito species by microscopy, abundance and composition, *P. falciparum* sporozoite rate, and PCR genotyping for insecticide resistance markers (*kdr g*ene) will be correlated to the epidemiological database to improve understanding of the malaria epidemiology and transmission in settings with varying malaria endemicities.

### Strengths and limitations

#### Strengths

This study is unique in that it aimed to investigate both the prevalence and transmission of malaria across multiple sites over time. A major strength of this study is the collection of both malaria epidemiological and entomological data over a four4-year period in different epidemiological settings, which may facilitate the generalisability of study findings across multiple contexts. The simultaneous collection of clinical, parasitological and entomological data will provide a unique opportunity for studying the effect of host, vector and environment interaction on malaria infection and disease patterns.

#### Limitations

This cohort study has several limitations. First, household (active surveillance) data were collected by structured questionnaires for events that occurred in the preceding six months, which may be subject to recall bias. To mitigate this bias, we used tested and validated tools based on prior national surveys, such as the DHSs, and well-trained, experienced interviewers. As data on acute illness were captured through the passive surveillance system self-reported, responses could also be validated.

Second, some participants moved out of the study area temporarily or permanently over the course of this longitudinal study, which may have led to bias or reduced statistical power. This limitation was expected, and the loss of follow-up was accounted for when determining the sample size for the cohort. Finally, some participants likely sought care for fever outside the study clinics, thus potentially limiting our ability to detect all acute malaria cases via passive surveillance. To minimise this bias, CHWs were trained to recognise malaria symptoms and encouraged participants to seek care at the study clinic. Additionally, the catchment area for participation was limited to 5 km, and participants were provided reimbursement for transportation. Despite these limitations, the data generated from this cohort will yield insights into malaria prevalence and transmission in Kinshasa Province, DRC and the country with the second highest malaria burden globally.

### Summary

This unique longitudinal study will provide malaria epidemiological and entomological data across diverse urban, periurban and rural settings of Kinshasa Province, DRC. The prospective data collected will allow us to examine a range of clinically relevant parameters, including the number of malaria episodes per participant and year (force of infection), time to symptomatic illness (natural history) and determinants of malaria prevalence and transmission, as well as detection of drug, diagnostic and insecticide resistance markers. Results from this study will improve our understanding of the malaria burden and transmission dynamics over time in settings with varying malaria endemicities and across age groups, thus providing vital information to improve the tailoring of malaria control strategies based on the level of malaria endemicity in the DRC. Additionally, the identification of determinants of infection can improve the targeting of interventions to improve control and, ultimately, eliminate malaria in the DRC.

### Collaboration

We welcome potential collaboration with other researchers. Researchers can contact the corresponding author for collaboration requests.

## supplementary material

10.1136/bmjopen-2024-085360 online supplemental file 1

10.1136/bmjopen-2024-085360 online supplemental file 2

10.1136/bmjopen-2024-085360 online supplemental file 3

## Data Availability

Data are available upon reasonable request.

## References

[1] WHO (2023). World malaria report 2022. https://www.who.int/teams/global-malaria-programme/reports/world-malaria-report-2023.

[2] DRC (2014). DHS report 2013-2014. https://www.unicef.org/drcongo/media/1046/file/COD-rapport-EDS.pdf.

[3] DRC (2021). PNLP. https://pnlprdc.org/wp-content/uploads/2022/09/Rapport-annuel-2021-des-activites-de-lutte-contre-le-Paludisme-1.pdf.

[4] Ilombe G, Matangila JR, Lulebo A (2023). Malaria among children under 10 years in 4 endemic health areas in Kisantu Health Zone: epidemiology and transmission. Malar J.

[5] Bigirinama RN, Ntaongo JA, Batumbo D (2020). Environmental and anthropogenic factors associated with increased malaria incidence in South‐Kivu Province, Democratic Republic of the Congo. Tropical Med Int Health.

[6] DRC (2007). Demographic and health survey 2007: dhs 2007 drc key findings-english.

[7] DRC (2014). Demographic health survey 2013-2014. https://www.dhsprogram.com/pubs/pdf/SR218/SR218.pdf.

[8] Institut National de la Statistique (INS) (2019). Enquête par grappes à indicateurs multiples, 2017-2018, rapport de résultats de l’enquête. (MICS6 Final Report-French).

[9] Ntamabyaliro NY, Burri C, Lula YN (2021). Knowledge of Antimalarials and Health Seeking Behaviour of Households in Case of Suspected Malaria in Democratic Republic of the Congo. Trop Med Infect Dis.

[10] Walldorf JA, Cohee LM, Coalson JE (2015). School-Age Children Are a Reservoir of Malaria Infection in Malawi. PLoS ONE.

[11] Cohee LM, Opondo C, Clarke SE (2020). Preventive malaria treatment among school-aged children in sub-Saharan Africa: a systematic review and meta-analyses. Lancet Glob Health.

[12] Simpson SV, Nundu SS, Arima H (2023). The diversity of Plasmodium falciparum isolates from asymptomatic and symptomatic school-age children in Kinshasa Province, Democratic Republic of Congo. Malar J.

[13] Tiono AB, Guelbeogo MW, Sagnon NF (2013). Dynamics of malaria transmission and susceptibility to clinical malaria episodes following treatment of Plasmodium falciparum asymptomatic carriers: results of a cluster-randomized study of community-wide screening and treatment, and a parallel entomology study. BMC Infect Dis.

[14] Doumbe-Belisse P, Kopya E, Ngadjeu CS (2021). Urban malaria in sub-Saharan Africa: dynamic of the vectorial system and the entomological inoculation rate. Malar J.

[15] Gari T, Kenea O, Loha E (2016). Malaria incidence and entomological findings in an area targeted for a cluster-randomized controlled trial to prevent malaria in Ethiopia: results from a pilot study. Malar J.

[16] SDG Target 3.3 communicable diseases (who.int). https://www.who.int/data/gho/data/themes/topics/sdg-target-3_3-communicable-diseases.

[17] Richards SL, Byrd BD, Reiskind MH (2020). Assessing Insecticide Resistance in Adult Mosquitoes: Perspectives on Current Methods. Environ Health Insights.

[18] Taylor SM, Antonia AL, Parobek CM (2013). Plasmodium falciparum sulfadoxine resistance is geographically and genetically clustered within the DR Congo. Sci Rep.

[19] Thomson R, Parr JB, Cheng Q (2020). Prevalence of *Plasmodium falciparum* lacking histidine-rich proteins 2 and 3: a systematic review. Bull World Health Organ.

[20] François R, Kashamuka MM, Banek K (2023). Plasmodium falciparum with pfhrp2/3 Deletion Not Detected in a 2018-2021 Malaria Longitudinal Cohort Study in Kinshasa Province, Democratic Republic of the Congo. Am J Trop Med Hyg.

[21] Sendor R, Banek K, Kashamuka MM (2023). Epidemiology of Plasmodium malariae and Plasmodium ovale spp. in Kinshasa Province, Democratic Republic of Congo. Nat Commun.

[22] Mwandagalirwa MK, Levitz L, Thwai KL (2017). Individual and household characteristics of persons with Plasmodium falciparum malaria in sites with varying endemicities in Kinshasa Province, Democratic Republic of the Congo. Malar J.

[23] Carrel M, Kim S, Mwandagalirwa MK (2021). Individual, household and neighborhood risk factors for malaria in the Democratic Republic of the Congo support new approaches to programmatic intervention. H Place.

[24] RDC (2020). Plan strategies national de lutte contre le paludisme.

[25] Namango IH, Marshall C, Saddler A The centres for disease control light trap (cdc lt) and the human decoy trap (hdt) compared to the human landing catch (hlc) for measuring anopheles biting in rural tanzania. In Review.

[26] Harris PA, Taylor R, Minor BL (2019). The REDCap consortium: building an international community of software platform partners. J Biomed Inform.

[27] Harris PA, Taylor R, Thielke R (2009). Research electronic data capture (REDCap)--a metadata-driven methodology and workflow process for providing translational research informatics support. J Biomed Inform.

