# Supplementary material for: Baseline characteristics for phase II of the Kinshasa Malaria Cohort Study: cohort profile
Source: BMJ Open. 2024 Nov 27;14(11):e085360. doi: 10.1136/bmjopen-2024-085360 (PMC11603822; doi:10.1136/bmjopen-2024-085360)
Supplement: online supplemental file 1 [file bmjopen-14-11-s001.pdf]

Supplement 1

Supporting document: Comparison of Phase I and Phase II study population characteristics

Study Population		**Phase I** 2015 -2017		**Phase II** 2018 - 2022		
Total population		1591		1635		
		n	%	n	%	**p-value**
**Age**						
	<5	366	23%	284	17%	0.000
	5-14	495	31%	530	32%	0.427
	15+	730	46%	821	50%	0.014
**Sex**						
	Male	713	45%	741	45%	0.773
	Female	878	55%	894	55%	0.773
**Health_Areas**						
	Bu	675	42%	586	36%	0.000
	Kimpoko	528	33%	588	36%	0.097
	Voix du Peuple	388	24%	461	28%	0.014
**Sites**						
	Bu	209	13%	196	12%	0.325
	Impuru	217	14%	200	12%	0.234
	Pema	249	16%	190	12%	0.001
	Ngamanzo	268	17%	250	15%	0.229
	Iye	92	6%	117	7%	0.113
	Kimpoko	168	11%	221	14%	0.010
	Voix du Peuple	388	24%	461	28%	0.014

We used R to perform two-sample proportion tests in key participant characteristics to test for differences between the two phases. The test results indicated some statistically significant but small differences (0%-6%) between Phase I’s and Phase II’s populations. Migration and aging populations influenced differences in age and study sites. The site differences drove the differences found at the health area level.
