# Supplementary material for: Baseline characteristics for phase II of the Kinshasa Malaria Cohort Study: cohort profile
Source: BMJ Open. 2024 Nov 27;14(11):e085360. doi: 10.1136/bmjopen-2024-085360 (PMC11603822; doi:10.1136/bmjopen-2024-085360)
Supplement: online supplemental file 2 [file bmjopen-14-11-s002.pdf]

Supplement 2: Household sampling and replacement

The sampling protocol for the enrollment of households into the longitudinal cohort study occurred with the Phase 1 study in 2017, and continued for Phase 2, and was designed to ensure that the full extent of each site was covered. This was accomplished as follows.

- Each site consisted of households built along a main road that bisected the site (see **Supplement 3**).
- To enroll households, we selected geographic coordinates at each end of each site, representing starting and finishing coordinates, and stored these coordinates in GPS units provided to the field team.
- The field team then enrolled households by walking the length of each site, beginning with the household nearest the starting coordinates and ending at the household nearest the finishing coordinates.

Dropout has the potential to diminish the spatial coverage within a given site. To maintain coverage in the event that a household drops out of the study, we enrolled the nearest neighboring unenrolled household. In the event that all neighboring households were unenrolled and equidistant from the household that dropped out, the household to the right (facing the dropout household) of the one that dropped out was selected. In the event that this household chose not to enroll in the study, the household to the left was selected. The field team iterated this process (i.e. switching from right to left) until a replacement household was enrolled.

Entomology sampling: As with the household sampling in the overall study, household sampling for entomological surveillance was done to ensure a spatially representative coverage of the households enrolled at each site. Specifically, using the GPS coordinates and household IDs of enrolled households, each site was divided into 5 groups with an equal number of households in each group, and then one household from each group was randomly selected to participate in entomological surveillance. If the household declined to enroll, it was replaced by the next closest household in that group.

Each group was constructed such that the households in each group are more proximate to one another than to other households, under the constraint that each group must contain an equal number of households (**Supplement3**). The procedure described ensures that (1) the entire village/quartier is covered by entomological surveillance and (2) that each group has a sufficient number of households to allow for alternative households to be selected should the first decline to enroll.
