# Supplementary material for: Baseline characteristics for phase II of the Kinshasa Malaria Cohort Study: cohort profile
Source: BMJ Open. 2024 Nov 27;14(11):e085360. doi: 10.1136/bmjopen-2024-085360 (PMC11603822; doi:10.1136/bmjopen-2024-085360)

Supplement 3: Example of household and Entomological Sampling Design.

As an example, Presented is a theoretical site of 15 house in a village/quartier. For household sampling, the boundaries of the village were mapped, and household enrollment started on one side of the village (green dot). The team moved along the road enrolling houses on either side until the end of the village (blue dot). For entomological sampling, the 15 households were divided into 5 groups of 3 households each (represented by the different colored boxes). Households were grouped together with other nearby household where possible. In some instances groups were close to other groups. For example, in the figure below households BCD are more proximate to one another than ABC, but the constraint that household A must be in a group with 2 nearby households leads to the grouping ABC instead of BCD, since otherwise household A would have no meaningful group assignment.

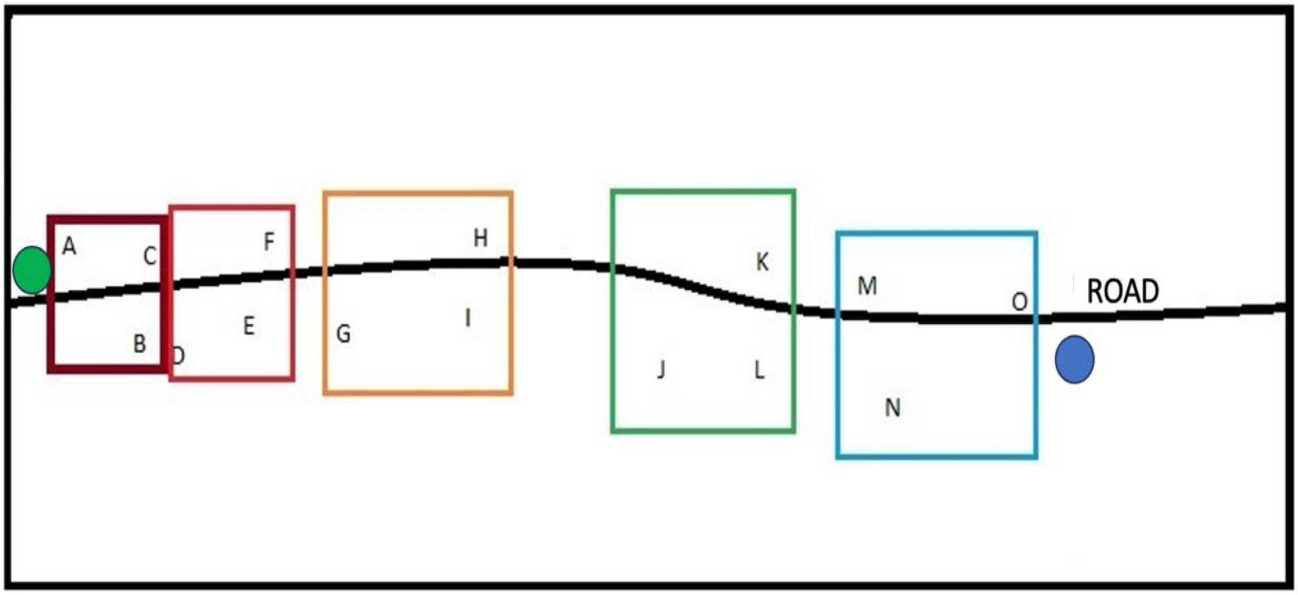

Supplement: online supplemental file 3 [file bmjopen-14-11-s003.pdf]
