# Supplementary material for: Baseline characteristics for phase II of the Kinshasa Malaria Cohort Study: cohort profile
Source: BMJ Open. 2024 Nov 27;14(11):e085360. doi: 10.1136/bmjopen-2024-085360 (PMC11603822; doi:10.1136/bmjopen-2024-085360)
Supplement: Supplementary file 4 [file bmjopen-2024-085360.reviewer_comments.pdf]

PEER REVIEW HISTORY

BMJ Open publishes all reviews undertaken for accepted manuscripts. Reviewers are asked to complete a checklist review form and are provided with free text boxes to elaborate on their assessment. These free text comments are reproduced below.

ARTICLE DETAILS

Title (Provisional)

Cohort Profile: Baseline Characteristics for Phase II of the Kinshasa Malaria Cohort Study

Authors

Kashamuka, Melchior Mwandagalirwa; Banek, Kristin; White, Samuel J.; Atibu, Joseph Losoma; Mazangama Nono, Mvuama M.; Bala, Joseph Alexandre Mavungu; Nseka, Tommy Mambulu; Kihuma, Georges; Diallo, Alpha Oumar; Nkalani, Marthe; Mahilu, Georges Emo; Manenga, Faustin; Varun, Goel; Vulu, Fabien; Mampuya, Pitshou Nsongo; Dinglasan, Rhoel; Bobanga, Thierry; Emch, Michael; Juliano, Jonathan J.; Parr, Jonathan; Mwapasa, Victor; Tshefu Kitoto, Antoinette

---

VERSION 1 - REVIEW

**Reviewer**	**1**
**Name**	**Patrick, Sean**
**Affiliation**	**University of Pretoria Faculty of Health Sciences, School of Health Systems and Public Health**
**Date**	**25-Feb-2024**
**COI**	**None**

This manuscript reports on a cohort profile: participant characteristics for phase II of the Kinshasa Malaria Cohort Study

Title

Suggested title: Cohort Profile: Baseline Characteristics of Phase II of the Kinshasa Malaria Cohort Study Participants

Introduction

The introduction section covers the basic information. What is missing is a reference to the SDGs which have malaria elimination as SDG3.3, a reference to the WHO World Malaria report giving malaria morbidity and mortality rates to place the study in context. Reference 1 should be updated as the 2023 World Malaria Report has already been published.

Cohort Study

Were the sites selected based on the settings or have there been high malaria cases reported in these areas pre-2018 that inform the specific site selection? The information in the cohort description should be included in the paragraph above. Example: We conducted the study in seven villages (study sites) located in three health areas in Kinshasa Province, selected in phase 1 (reference 20, 21)

The authors should provide more detail on the loss to follow up in the rainy season in Mar/Apr 2020, then an increase in participants in Mar/Apr 2021.

Community engagement

Can the authors provide a sentence or two on the sensitization referred to in this section?

The Cohort profile is well presented and the reasoning for the site selection has already been published. I would have appreciated a paragraph discussing the data presented in Tables 2 and 3, offering some views, since this is phase II of the study. For example, the high unemployment rate possibly contributes to participants moving or missing follow-up visits due to job seeking?

**Reviewer**	**2**
**Name**	**Sumari, Deborah**
**Affiliation**	**Ifakara Health Institute, Biomedical Research and Clinical Trials**
**Date**	**06-Mar-2024**
**COI**	**I have no competing interests**

A good longitudinal study but statistical calculations conducted need to be shown and how the sample size was obtained from this particular study. Study findings should be visibly discussed and concluded. The authors indicated there are future plans, they need to clearly indicate if there is going to be a follow up study based on the current findings from this study or is just a recommendation for future research.

NOTE: The reviewer provided a marked copy with additional comments. Please contact the publisher for full details

---

VERSION 1 - AUTHOR RESPONSE

Responses to reviewers

Below is a point-by-point response to the reviewers' comments and concerns.

Editor's Comments

1) Please revise the ‘Strengths and limitations of this study’ section of your manuscript (after the abstract). This section should contain up to five short bullet points, no longer than one sentence each, that relate specifically to the methods. The novelty, aims, results or expected impact of the study should not be summarized here.

Response: We have noted this suggestion and have revised the “strengths and limitations” (page 2 lines 30-37)

Reviewer 1 Comments

1) Suggested title: Cohort Profile: Baseline Characteristics of Phase II of the Kinshasa Malaria Cohort Study Participants

Response: Thank you for your title suggestion to replace “Participant” with “Baseline.” As the content of the manuscript is mainly focused on details related to the baseline, we agree with the comment and have changed the title.

2) The introduction section covers the basic information. What is missing is a reference to the SDGs which have malaria elimination as SDG3.3, a reference to the WHO World Malaria report giving malaria morbidity and mortality rates to place the study in context. Reference 1 should be updated as the 2023 World Malaria Report has already been published.

Response: Thank you for your comment. We have updated the World Malaria Report reference and have added to the introduction a reference for the SDG 3.3 communicable diseases (WHO.int), (page 3, lines 79-81). The World Malaria Report reference on page 19, (lines 484-486), has been updated. The reference for the SDG 3.3 is on page 21, (lines 547-549).

3) Were the sites selected based on the settings or have there been high malaria cases reported in these areas pre-2018 that inform the specific site selection? The information in the cohort description should be included in the paragraph above. Example: We conducted the study in seven villages (study sites) located in three health areas in Kinshasa Province, selected in phase 1 (reference 20, 21)

Response: Thank you for your comment. Accordingly, we have added “selected in phase 1” on page 4, (line 99). In addition, this study is a continuation of a previous study, and the rationale for site selection has been previously described (Reference 22 on page 21-22, lines 573-577 and reference 23 on page 22, lines 578-583).

4) The authors should provide more detail on the loss to follow up in the rainy season in Mar/Apr 2020, then an increase in participants in Mar/Apr 2021.

Response. We agree with the reviewer’s comments. Accordingly, we have added the following text to page 10, (lines 298-301)

“It should be noted that movement restrictions due to the COVID-19 pandemic impacted data collection; thus, the active household surveillance visit was not conducted in Lingwala in March-April 2020, as shown in the study overview(figure 2)”.

5) Community engagement. Can the authors provide a sentence or two on the sensitization referred to in this section?

Response: We agree with the reviewers’ request. Accordingly, we have added text on page 9-10 (lines 272-284)

6) The Cohort profile is well presented and the reasoning for the site selection has already been published. I would have appreciated a paragraph discussing the data presented in Tables 2 and 3, offering some views, since this is phase II of the study. For example, the high unemployment rate possibly contributes to participants moving or missing follow-up visits due to job seeking?

Response: Thank you for this suggestion. However, this is out of the scope of this type of manuscript as defined in the notes to authors. Cohort Profiles do not present study findings. Results will be presented in subsequent papers. However, we have offered some views in the baseline findings section: high net used in the household in Kimpoko, probably due to high mosquito nuisance (page 13, table 2); high loss to follow-up in urban site (figure 3), due probably to high unemployment, (page 14, table 3); low percentage of less than 5 years age category in the urban site due to probably to high education and high health index in the urban site,(page 14, table 3). We have also included a supplemental Table 1 describing Phase I and II cohort characteristics and similarities. In terms of reasons for migration and

population movement, we only asked if they moved, not the reason why there was movement.

Reviewer 2 Comments

1) A good longitudinal study, but statistical calculations conducted need to be shown and how the sample size was obtained from this particular study.

Response: Thank you for this comment. We have added a paragraph describing how Statistical calculation and sample size have been calculated on page 5 (lines 128-131).

"Using an estimated malaria prevalence of 20%, participant loss-to-follow-up of 15%, and 90% power to detect an increase in prevalence to 25% (alpha=0.05) when comparing outcomes between time points, the target sample size was estimated to be 1600."

We have also provided a detailed description of the household and entomological sampling design in supplement 2.

2) Study findings should be visibly discussed and concluded.

Response: Thank you for this suggestion. However, this seems out of the scope of this type of manuscript as Cohort Profiles do not present study findings as outlined in the BMJ Open notes to authors. Detailed results will be presented in subsequent papers. However, simple baseline results related to patient characteristics and outcomes are presented in the "Baseline Findings" section pages 10-12.

3) The authors indicated there are future plans, they need to clearly indicate if there is going to be a follow up study based on the current findings from this study or is just a recommendation for future research.

Response: Yes, this study has provided data for several papers and has served as the foundation for a number of concurrent and subsequent studies, including a Phase III follow-up study.

Additional clarifications or edits

1) We have crosschecked all of the figures throughout the document and have edited it for consistency.

2) The author list remains unchanged, but we have updated the author affiliations.

3) In addition to the above comments, all spelling and grammatical errors pointed out by the reviewers have been corrected.

VERSION 2 - REVIEW

**Reviewer**	**2**
**Name**	**Sumari, Deborah**
**Affiliation**	**Ifakara Health Institute, Biomedical Research and Clinical Trials**
**Date**	**10-Sep-2024**
**COI**	**None**

The authors have responded to the comments as requested by the reviewers.
